# Consumption of a Diet Rich in Cottonseed Oil (CSO) Lowers Total and LDL Cholesterol in Normo-Cholesterolemic Subjects

**DOI:** 10.3390/nu4070602

**Published:** 2012-06-26

**Authors:** Kathleen E. Davis, Chandan Prasad, Victorine Imrhan

**Affiliations:** Nutrition and Food Sciences, Texas Woman’s University, PO Box 425888, Denton, TX 76204, USA; Email: KDavis10@twu.edu (K.E.D.); CPrasad@twu.edu (C.P.)

**Keywords:** cardiovascular disease, cholesterol, cottonseed oil, lipid profile, triglycerides

## Abstract

Animal data indicates that dietary cottonseed oil (CSO) may lower cholesterol; however, the effects of a CSO**-**rich diet have not been evaluated in humans. Thirty-eight healthy adults (aged 18%#x2013;40; 12 males, 26 females) consumed a CSO rich diet (95 g CSO daily) for one week. Anthropometric measurements were obtained, and blood was drawn pre- and post-intervention. Serum lipids (total cholesterol (TC), high density lipoprotein (HDL), low density lipoprotein (LDL), triglyceride (TG), and free fatty acids (FFA)) were assayed. There was no change in weight or waist circumference among participants. There was no change in HDL (Pre: 1.27 ± 0.4 mmol/L; Post: 1.21 ± 0.3 mmol/L) or TG (Pre: 0.91 ± 0.6 mmol/L; Post: 1.06 ± 1.0 mmol/L). Total cholesterol and LDL were reduced (TC Pre: 4.39 ± 0.9 mmol/L; Post: 4.16 ± 0.8 mmol/L; LDL Pre: 2.70 ± 0.8 mmol/L; Post: 2.47 ± 0.6 mmol/L). When data were grouped by sex, total cholesterol was reduced in female participants (Pre: 4.34 ± 0.9 mmol/L; Post: 4.09 ± 0.8 mmol/L). Consumption of a high fat, CSO-rich diet for one week reduced total cholesterol in female participants without reducing HDL.

## 1. Introduction

Hypercholesterolemia, particularly increases in LDL-cholesterol, have been clearly associated with increased risk for cardiovascular disease [1,2]. In strategies for prevention of hypercholesterolemia and subsequent management of cardiovascular disease, modification of body mass index (BMI), diet, and lifestyle have important roles to play [1]. The literature suggests a differential role of types of oil and fat in diet on plasma total cholesterol and its components (HDL and LDL) [1,2,3,4,5].

Cottonseed oil (CSO) is a common vegetable oil used in a variety of foods in the United States and worldwide. Its consumption throughout the world has remained relatively steady over the past 5 years with approximately 4.8 million metric tons consumed in 2006/2007 and 4.6 in 2009/2010. This is higher than consumption of olive oil (2.9 million metric tons) [6]. In the US, consumption of CSO ranks behind corn oil, soybean oil, and canola oil [6], but it is used in the manufacture of potato chips and baked goods and may be used in some margarines and salad dressings. Despite its common uses in food production, its role in lipid metabolism in humans remains largely unknown [7]. 

Animal studies by Radcliffe and Narins [7,8] have indicated that replacement of corn oil with CSO for four weeks decreased total cholesterol along with HDL in rats. The lipid lowering effects of CSO are present in spite of its relatively high concentrations of saturated fatty acids compared to corn oil (26% saturated fat compared to 13%) and lower concentrations of monounsaturated fats (20% compared to 28%). CSO has a fatty acid profile that is 26% saturated fat, 20% monounsaturated fat, and 55% polyunsaturated fat. Stearic acid accounts for 3% of the total fatty acid content, palmitic acid 22%, and linoleic acid 54% [9]. Another study by Radcliffe and Narins showed that rats fed CSO had increased adipose tissue levels of saturated fatty acids [10]. Given that saturated fats are well known to be associated with atherogenic lipid profiles, these separate findings are somewhat puzzling. Radcliffe *et al*. have hypothesized that the lipid lowering effects of CSO may be due to the nonsaponifiable portion of this oil [7], which contains alpha-tocopherol and beta sitosterol. 

The present study was undertaken to determine the effects of short-term consumption of a CSO-rich diet on serum lipids in healthy adults.

## 2. Methods

This study was approved by the Texas Woman’s University Institutional Review Board’s Human Subjects Committee, and each participant provided written consent prior to participation in the study. Twelve men (Age: 26.8 ± 4.7 years; BMI: 24.2 ± 2.1 kg/m^2^) and twenty-seven women (Age: 24.2 ± 4.9 years; BMI: 24.2 ± 3.1 kg/m^2^) were recruited. One female participant dropped out due to inability to complete the blood draw. Thus, 38 participants (12 males, 26 females) were included in the study data. 

Male and female participants were included in the study if they were ages 18%#x2013;40 and not taking medication which could influence lipid levels. Exclusion criteria related to health concerns included self-reported hypertension, high cholesterol, kidney disease, diabetes, lung disease, heart disease, and liver disease. Smokers were also excluded.

Participants were recruited from the university campus community, including staff and students. The majority of participants were students and/or friends of students. Participants were instructed to adhere to their usual level of activity during the study period, and information on any supplements or medications taken by participants was also obtained. No participants were taking supplements other than a multivitamin/mineral supplement, calcium, vitamin C or a B complex. No participants were taking medication other than birth control pills.

### 2.1. Participants

Potential participants were advised to report to the research location on a Saturday morning following a twelve hour fast. Weight was measured to the nearest 0.1 pound using a Tanita electronic scale, and height was measured to the nearest 0.25 inch using a stadiometer. Waist circumference was measured to the nearest 0.25 inch at the level of the hip bone using two consecutive measures with a non-elastic tape. Measurements were converted to metric units (kilograms, centimeters) for reporting purposes.

### 2.2. Diet, Blood Collection and Analyses

After anthropometric measurements were obtained, a 24 h dietary recall was completed by an experienced registered dietitian using the USDA Multiple Pass Method [11,12,13] in order to establish a baseline for usual diet. Fasting blood was obtained, allowed to clot for 30 min, centrifuged at 3600 rpm for 15 min to obtain serum, and stored at −80 °C. 

Then participants were provided with a breakfast of 6 ounces of 100% apple juice and three carrot muffins made with cottonseed oil (7 g per muffin). Participants were advised to follow their usual diet for the remainder of the study days *except* they were given five muffins and one six-ounce 100% apple juice box to consume for breakfast and snacks daily Monday through Friday the following week. 25 muffins (5 daily for 5 days) were sent home with each participant. In addition, participants consumed lunch within the laboratory Monday through Friday. The study lunch diet consisted of 57 g of chicken strips, 57 g of fried potatoes, and one milk shake (all prepared with CSO: 60 g per meal). Dinner and snacks were consumed at the discretion of the participants. Any muffins not consumed were requested to be returned to the researchers. In total the study diet contained 95 g per day of CSO. In order to help determine adherence to the study protocol participants were also instructed to complete a five day food diary during the study period. 

The following Saturday, participants returned to the laboratory after a twelve hour fast for a final round of anthropometric measurements and blood draws as described. At that time food records were reviewed with participants, and clarification was obtained on any incomplete items. Food records were found to be very thorough. 

Twenty four hours dietary recall data on baseline diet and five day food record dietary intake data related to the study diet were analyzed using Nutrition Data System for Research software version 2009 developed by the Nutrition Coordinating Center (NCC), University of Minnesota, Minneapolis, MN. Both 24 h recalls and five day food records were evaluated for kilocalories, protein, carbohydrate, fiber, total, saturated, polyunsaturated, monounsaturated, and trans fat and cholesterol, vitamins, and minerals.

The amount of cottonseed oil tested in this study was determined based on the constraints of the feeding facility, which made a plan to give breakfast, lunch, and snacks to the participants most feasible. Recipes were identified, revised to include CSO, and tested for acceptability. When the oils were replaced based on these guidelines, the study diet contained 95 g CSO. This was felt to be a large enough dose to effect minor changes in metabolic indicators even in a short time period. The one-week intervention was chosen in order to pilot the acceptability of the diet and get maximum participation from study subjects. Despite the high level of fat from CSO consumed by the participants in the study, all participants finished most muffins and study foods. Two participants did not consume one to two muffins on one study day but were deemed to qualify as compliant. One participant complained of nausea and abdominal discomfort during the study. Others enjoyed the food and did not complain of discomfort. Adherence to the regimen was extremely high as evidenced by review of the five day food records, discussion with the participants and lack of returned food items. 

Sera were analyzed for TG, TC, HDL, and total free fatty acids (FFA) using commercially available kits (StanBio, Boerne, TX; Biovision, Mountain View, CO). LDL was calculated using the Friedewald Equation [LDL-chol] = [Total chol] − [HDL-chol] − [TG]/5. 

### 2.3. Statistical Analyses

Data were analyzed for normality, and potential numerical outliers identified using box plots and 1.5 times the Q_1_ to Q_3_ interquartile range. Paired *t*-tests were used to compare pre and post levels for serum lipids and anthropometrics: weight, height, BMI, and waist circumference. Data were analyzed with and without outlier data for comparison. Correlations between pre and post weight, BMI, and waist circumference, and change in weight with each serum parameter were also done. In addition, change in fasting cholesterol was correlated with all study parameters, including change in dietary intake between baseline and study diet. Statistical analyses were performed using SPSS version 19.0 for Windows.

## 3. Results

Analysis of the baseline diet of participants based on the 24 h recall (Table 1) revealed lower baseline energy intake for males and females compared to the study diet. Consequently there were significant differences in consumption of total fat, carbohydrate and polyunsaturated fatty acids for males and females (*p* < 0.01). The diets were significantly different in saturated fat content for females (*p* = 0.05). It was also observed that females consumed a 28% fat, 57% carbohydrate diet at baseline whereas males in the study consumed 36% of calories as fat and 45% as carbohydrates. The study diet and baseline diets were similar in percentage of calories from saturated fat although during the study participants consumed a higher net amount of saturated fat. Both females and males in the study consumed higher total amount and percentage of the diet as PUFA during the study compared to baseline. Trans fat consumption did not differ much in amount or percentage. Both males and females consumed more dietary cholesterol during the feeding study.

Dietary intake among participants during the study period was also assessed. Male participants had average daily energy intake of 14,175 ± 2334 kJ. Average caloric intake for female participants was 10,623 ± 1418 kJ (Table 2). Carbohydrates comprised 46 and 49% of calories in men and women respectively. Fat contributed 39.5 and 39% of calories in men and women. Dietary fiber intake was approximately 22 g daily in men and 19 g in women. Mean cholesterol intake in men was 335 ± 108 mg daily. In women, average cholesterol intake was 202 ± 50 mg.

**Table 1:** Baseline Dietary Intake of Participants ^1^.

Nutrient	Males	Percentage of KJ	Females	Percentage of KJ
Energy (KJ/day)	9360 ± 3090	--	7527 ± 2517	--
Total Fat (g/day)	90 ± 39	36%	57 ± 35	28%
Saturated Fat (g/day)	32 ± 21	12.8%	19 ± 11	9.6%
Trans Fat (g/day)	3 ± 2	1.1%	2 ± 1	0.8%
MUFA (g/day)	31 ± 13	12.5%	19 ± 12	9.5%
PUFA (g/day) ^2^	20 ± 9	8%	14 ± 13	7%
Carbohydrate (g/day)	255 ± 94	44.6%	255 ± 80.0	57%
Dietary fiber (g/day)	17 ± 7	--	20 ± 6	--
Protein (g/day)	94 ± 36	16.8%	74 ± 17	16.4%
Cholesterol (mg/day)	266 ± 142	--	164 ± 78	--

^1^ Mean ± SD (all such values); ^2^ PUFA includes *n*-6 only, not *n*-3 fatty acids.

**Table 2:** Nutrient Intake of Participants during the Study ^1^.

Nutrient	Males (*n* = 12)	Percentage of KJ	Females (*n* = 26)	Percentage of KJ
Energy (KJ/day)	14,175 ± 2334	--	10,623 ± 1418	--
Total Fat (g/day)	149 ± 34	39.5%	110 ± 19	39%
Saturated Fat (g/day)	44 ± 13	12%	30 ± 6	11%
Trans Fat (g/day)	3 ± 2	0.7%	1 ± 1	0.4%
MUFA (g/day)	40 ± 13	14.4%	25 ± 6	9.0%
PUFA (g/day) ^2^	54 ± 9	10.6%	47 ± 6	16.7%
Carbohydrate (g/day)	393 ± 56	46%	313 ± 53	49%
Dietary fiber (g/day)	22 ± 5	--	19 ± 4	--
Protein (g/day)	112 ± 23	13.2%	78 ± 14	12%
Cholesterol (mg/day)	335 ± 108	--	202 ± 50	--

^1^ Mean ± Standard Deviation (SD) (all such values); ^2^ PUFA includes *n*-6 only, not *n*-3 fatty acids.

There was no significant change in waist circumference following week-long consumption of a diet rich in CSO in male (Pre: 84.8 ± 7.9 cm; Post 84.6 ± 8.1 cm) or female (Pre: 76.9 ± 7.3 cm; Post: 76.7 ± 7.6 cm) participants (Table 3). In contrast, there was a very modest increase in body weight of male participants (Pre: 77.4 ± 11.2 kg; Post: 78.1 ± 11.1 kg; *p* = 0.05) but not female participants (Pre: 65.3 ± 9.5 kg; Post: 65.4 ± 9.5 kg) participants. 

**Table 3:** Anthropometrics of Participants before and after 5 Days of Cottonseed Oil-Rich Diet ^1^.

	Males & Females (*n* = 38)			Males (*n* = 12)			Females (*n* = 26)		
	Pre	Post	*p*-Value	Pre	Post	*p*-Value	Pre	Post	*p*-Value
**Weight (kg)**	69.1 ± 11.5	69.4 ± 11.5	0.12	77.4 ± 11.2	78.1 ± 11.1	0.05 *	65.4 ± 9.5	65.3 ± 9.5	0.60
**Waist (cm)**	79.5 ± 8.4	79.2 ± 8.4	0.46	84.8 ± 7.9	84.6 ± 8.1	0.63	77.1 ± 7.4	76.7 ± 7.6	0.59

^1^ Mean ± Standard Deviation (SD) (all such values).

The analysis of serum lipids in all participants showed a decrease in TC and LDL over the course of the study (TC Pre: 4.39 ± 0.9 mmol/L; Post 4.16 ± 0.8 *p* = 0.01; LDL Pre: 2.70 ± 0.8 mmol/L; Post: 2.47 ± 0.6 mmol/L; *p* = 0.03) with no change in HDL, TG, or total FFA (Table 4). When data were grouped by sex, TC was significantly reduced in females only (Pre: 4.34 ± 0.9 mmol/L; Post: 4.09 ± 0.8 mmol/L; *p* = 0.02). Males showed a similar trend which was non-significant (TC Pre: 4.50 ± 0.8 mmol/L; Post: 4.34 ± 0.8 mmol/L; *p* = 0.32) (Table 4). Calculated LDL showed a trend for lowering in females which was not significant (Pre: 2.63 ± 0.9 mmol/L; Post: 2.40 ± 0.7 mmol/L; *p* = 0.08). LDL in males showed a similar trend toward lowering but did not reach significance (Table 4). 

**Table 4:** Serum Profile of Participants before and after 5 Days of Cottonseed Oil-Rich Diet ^1^.

	Males & Females (*n* = 38)			Males (*n* = 12)			Females (*n* = 26)		
	Pre	Post	*p*-Value	Pre	Post	*p*-Value	Pre	Post	*p*-Value
**TC (mmol/L)**	4.39 ± 0.9	4.16 ± 0.8	0.01 *	4.50 ± 0.8	4.34 ± 0.8	0.32	4.34 ± 0.9	4.09 ± 0.8	0.02 *
**LDL (mmol/L)**	2.70 ± 0.8	2.47 ± 0.6	0.03 *	2.85 ± 0.8	2.60 ± 0.6	0.20	2.63 ± 0.9	2.40 ± 0.7	0.08
**HDL (mmol/L)**	1.27 ± 0.4	1.21 ± 0.3	0.16	1.08 ± 0.3	1.00 ± 0.3	0.4	1.36 ± 0.3	1.31 ± 0.30	0.28
**TG (mmol/L)**	0.911 ± 0.6	1.06 ± 1.0	0.18	1.25 ± 0.8	1.61 ± 1.4	0.25	0.75 ± 0.5	0.81 ± 0.6	0.51
**FFA (nmol/µ** **L** **)**	0.120 ± 0.07	0.106 ± 0.05	0.42	0.148 ± 0.12	0.108 ± 0.05	0.88	0.112 ± 0.05	0.106 ± 0.05	0.32

^1^ Mean ± Standard Deviation (SD) (all such values).

No significant correlations were identified which would link the change in cholesterol to any study diet parameter in the group as a whole using Pearson’s correlations. In addition, change in dietary intake parameters between baseline and study diet were not correlated with change in TC or any other serum parameter. However, cholesterol dietary intake in men was correlated positively with increase in TC during the study (*R* = 0.63, *p* = 0.03).

Change in energy intake did not correlate with weight change in the group as a whole. When participant data was grouped by sex, there was a positive relationship between energy intake and weight gain in women (*R* = 0.44, *p* = 0.02). However, it should be noted that in general men in the study had much higher energy intake during the study compared to baseline diet. This was also true for women but was more pronounced in men.

## 4. Discussion

Given the comparatively high saturated fatty acid profile and lower mono and polyunsaturated fatty acid profile of cottonseed oil, an atherogenic effect might be expected with increased consumption of this oil [9]. However, preliminary data from this pilot study indicate that the interaction of lipids in the diet may be more complex than fatty acid profile alone. A one-week feeding would not have been expected to make any impact on total cholesterol or LDL, but in this study, total cholesterol was reduced significantly in female participants. There was a similar lowering effect for male participants, but it did not reach statistical significance. This may be attributable to the small sample of males. It is also possible that the consumption of a high cholesterol diet among the male participants (352.4 ± 121.6 mg daily) may have confounded the cholesterol lowering ability of the CSO. This hypothesis is supported by the correlation data indicating that in men higher dietary cholesterol was associated with increasing TC (*R* = 0.63, *p* = 0.03). Because of the physical limitations of the feeding facility, providing only breakfast and lunch on campus was determined to be feasible at the time of the study. Thus, participants had some latitude in choosing their diet for dinner. It was observed that men in the study chose many fast food meals for dinner and for additional snacks.

The mechanism for the decrease in blood lipids is not known. It is possible that the non-saponifiable portion of the oil, which contains tocopherols and beta sitosterol [14], may be responsible for modifying the blood lipid response together with overall alteration of the dietary pattern due to the dietary intervention. The decrease is particularly surprising given that most participants increased their energy intake overall as well as their intake of total fat, polyunsaturated fat, among females, saturated fat, and among males, cholesterol. Because baseline diet was determined using 24 h recall, it is not likely to be as accurate an estimation of usual diet as the five day food record was of intake during the study period. Despite multiple passes, participants were suspected of under-reporting intake on the 24 h recall. However, based on the analysis, study participants seemed to have similar meals for dinner and some snacks on study days compared to the 24 h recall days. The primary difference in the diets was the addition of the study foods, which replaced some usually consumed foods. In addition, change in energy intake, macronutrient intake and other dietary components measured did not correlate with change in TC or any other serum parameter.

The composition of the participants’ diet appears to be atherogenic with total fat intake of 39% (a high fat diet) and carbohydrate intake of 48% of calories. In addition, saturated fatty acids provided 11% of calories, polyunsaturated fatty acids 16%, and monounsaturated fats 9.5% on average. This caused the average diet of participants to exceed current American Heart Association recommendations for saturated fat intake (currently less than 7% of total calories). However, monounsaturated fat intake is somewhat higher than is typical for Americans [15]. In spite of this sub-optimal dietary intake profile, serum cholesterol was still lowered. Dietary fiber intake was relatively low (19 g daily on average for females or only about 7 g/1000 calories, well below the recommendation of 14 g/1000 calories). Dietary fiber likely did not account for the decrease in cholesterol as evidenced by lack of any correlation between fiber intake and change in cholesterol. Weight loss did not occur in either group of participants and thus is also not responsible for the change in cholesterol.

The non-saponifiable portion of CSO contains beta sitosterol (a plant sterol) and alpha tocopherol. Beta sitosterol is a possible candidate for the reduction in blood lipids [15], but given that cottonseed oil provides about 3640 mg/g oil [11], the cottonseed oil portion of the study diet provided only about 0.35 g daily, which is well below the 2 g daily that was hypothesized by Law to be required for a cholesterol lowering effect [16,17]. However, Hendricks *et al*. found no statistically significant difference in the cholesterol lowering power of three doses of plant sterols: (0.83, 1.61 and 3.24 g daily). Each dose resulted in a reduction in total cholesterol of approximately 5%–10%. A lower dose of 0.35 g was not evaluated in that study. [18] A recent review found that there is a continuous dose dependent relationship between phytosterols and their cholesterol lowering effect with doses as low as 0.45 g and 0.8 g daily having a significant cholesterol lowering impact [19]. It is possible that increased dietary intake of tocopherol combined with increased dietary sterols and polyunsaturated fats led to the lipid lowering effect of the diet. It is also possible that the reduction in TC was simply due to chance. However, based on the highly significant *p*-value in the entire group (*p* = 0.01), the trend towards lowering in men, the very high dose of CSO given, and our experience with the animal data, we do not believe this to be the case.

## 5. Conclusions

We conclude that CSO may lower cholesterol effectively, possibly making it a good candidate for inclusion in margarines and shortening, where it originated. (A common brand of shortening in the United States is Crisco, an acronym for crystallized cottonseed oil, which was composed of CSO until cotton was needed in higher amounts for cloth during World War II. At that time, shortening began to be made with soybean oil in the USA). A larger, longer-term study and one including participants with elevated serum cholesterol is advisable before such recommendations can be made. In addition we would like to study the inclusion of cottonseed oil in more typical quantities in a diet over a period of time to determine if the effects are possible to achieve without such high levels of intake. 
